# Supplementary material for: Influence of Magnesium Oxide on the Structure and Catalytic Activity of the Wustite Catalyst for Ammonia Synthesis
Source: Materials (Basel). 2022 Nov 23;15(23):8309. doi: 10.3390/ma15238309 (PMC9739429; doi:10.3390/ma15238309)
Supplement: Supplementary file 1 [file materials-15-08309-s001.zip › materials-1953417-SI.pdf]

materials

Supplementary Material

Influence of Magnesium Oxide on the Structure and Catalytic Activity of the Wustite Catalyst for Ammonia Synthesis

Artur Jurkowski, Aleksander Albrecht, Dariusz Moszyński, Rafał Pelka and Zofia Lendzion-Bieluń *

Department of Inorganic Chemical Technology and Environment Engineering, Faculty of Chemical Technology and Engineering, West Pomeranian University of Technology in Szczecin, Piastów Ave. 42, 71-065 Szczecin, Poland
* Correspondence: Zofia.Lendzion-Bielun@zut.edu.pl

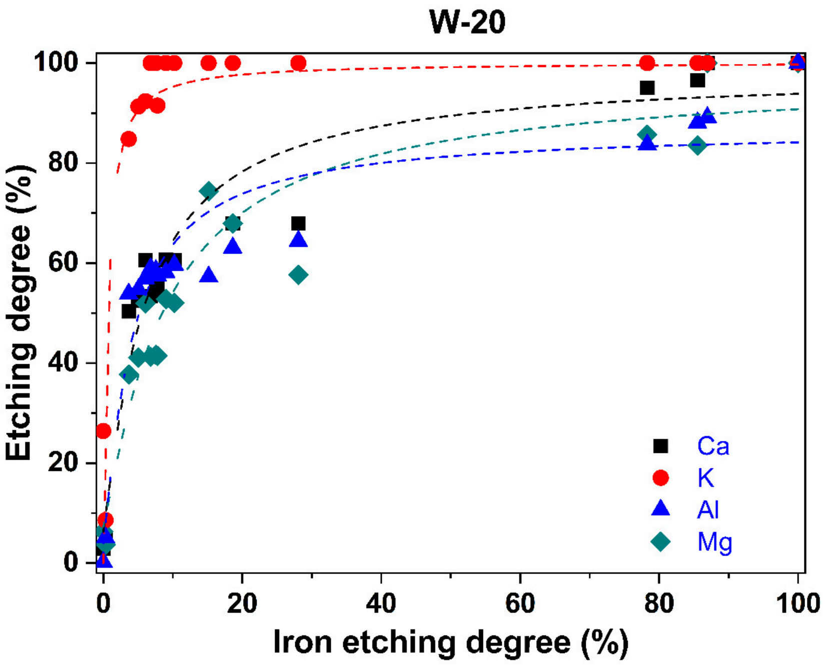

Figure S1. The degree of promotors etching related to the degree of iron etching in the precursor W-20.

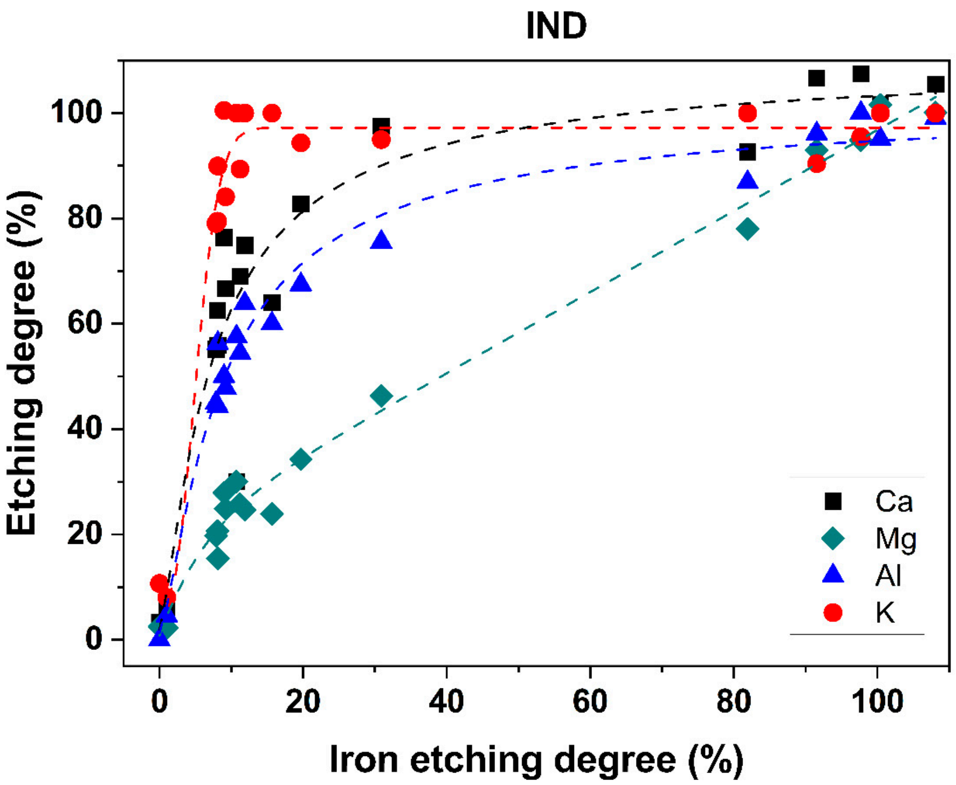

Figure S2. The degree of promotors etching related to the degree of iron etching in the precursor IND.

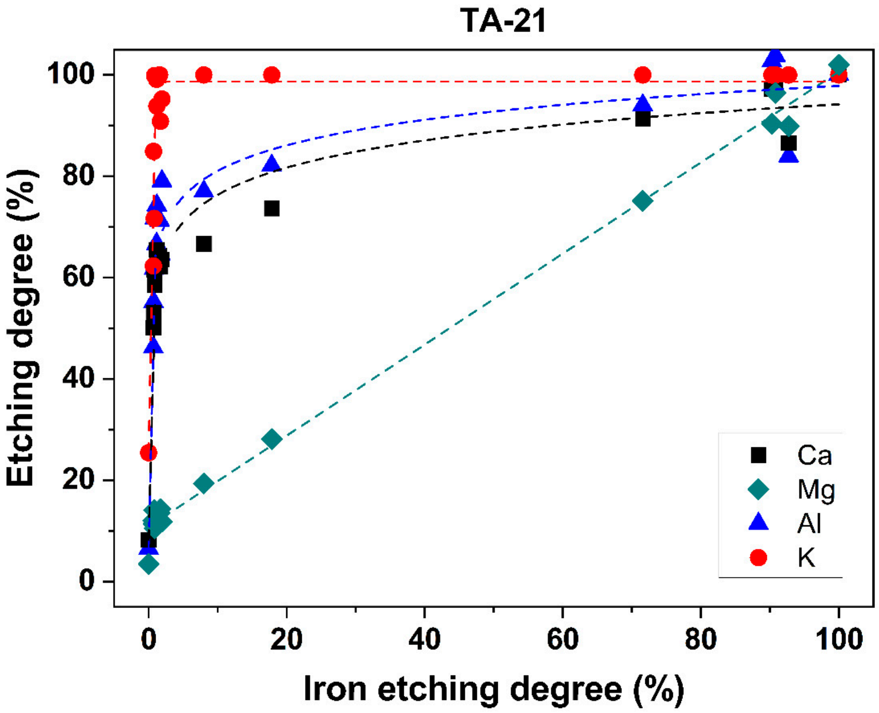

Figure S3. The degree of promotors etching related to the degree of iron etching in the precursor TA-21.

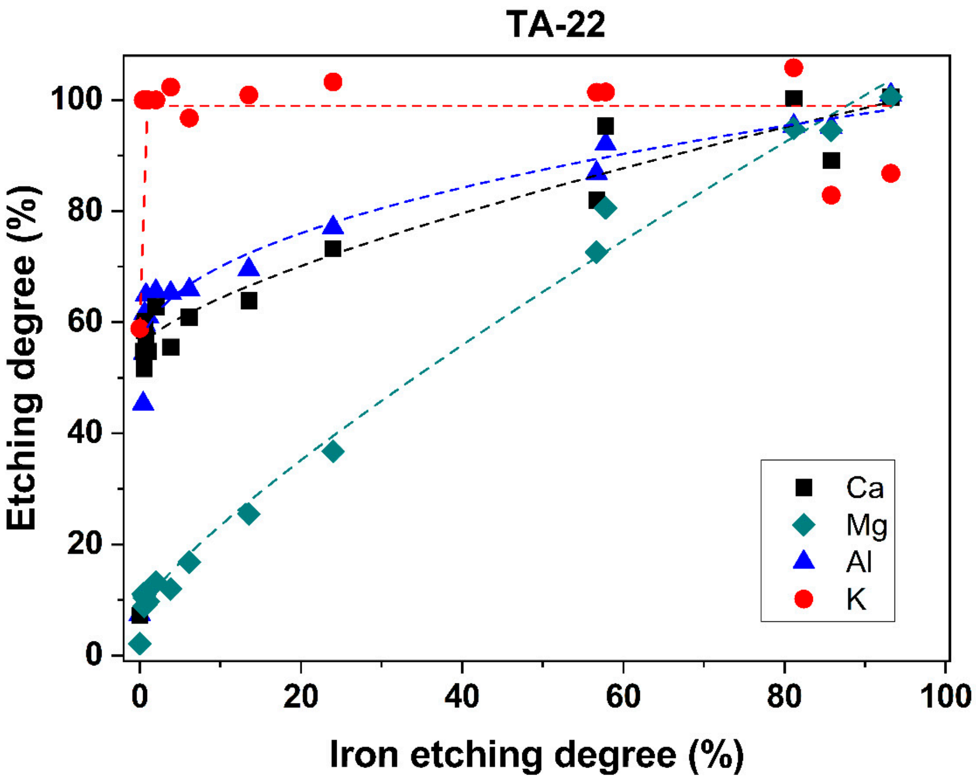

Figure S4. The degree of promotors etching related to the degree of iron etching in the precursor TA-22.

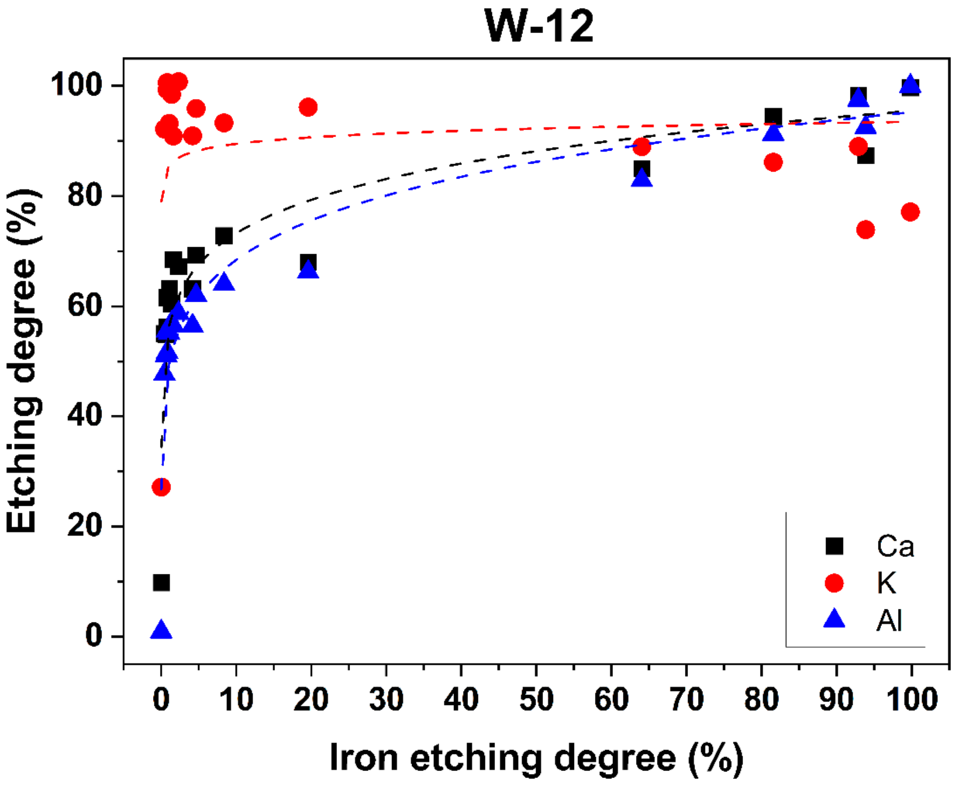

Figure S5. The degree of promotors etching related to the degree of iron etching in the precursor W-12.
